# Flow of Non-Newtonian Fluids in a Single-Cavity Microchannel

**DOI:** 10.3390/mi12070836

**Published:** 2021-07-18

**Authors:** Mahmud Kamal Raihan, Purva P. Jagdale, Sen Wu, Xingchen Shao, Joshua B. Bostwick, Xinxiang Pan, Xiangchun Xuan

**Affiliations:** 1 Department of Mechanical Engineering, Clemson University, Clemson, SC 29634-0921, USA; mraihan@clemson.edu (M.K.R.); purvaj@clemson.edu (P.P.J.); senw@clemson.edu (S.W.); jbostwi@clemson.edu (J.B.B.); 2 College of Marine Engineering, Dalian Maritime University, Dalian 116026, China; dmupanxx@gmail.com; 3 Department of Chemical and Biomolecular Engineering, Johns Hopkins University, Baltimore, MD 21218, USA; xshao8@jhu.edu; 4 Maritime College, Guangdong Ocean University, Zhanjiang 524088, China

**Keywords:** polymer solution, viscoelasticity, shear thinning, inertia, microfluidic model, porous media

## Abstract

Having a basic understanding of non-Newtonian fluid flow through porous media, which usually consist of series of expansions and contractions, is of importance for enhanced oil recovery, groundwater remediation, microfluidic particle manipulation, etc. The flow in contraction and/or expansion microchannel is unbounded in the primary direction and has been widely studied before. In contrast, there has been very little work on the understanding of such flow in an expansion–contraction microchannel with a confined cavity. We investigate the flow of five types of non-Newtonian fluids with distinct rheological properties and water through a planar single-cavity microchannel. All fluids are tested in a similarly wide range of flow rates, from which the observed flow regimes and vortex development are summarized in the same dimensionless parameter spaces for a unified understanding of the effects of fluid inertia, shear thinning, and elasticity as well as confinement. Our results indicate that fluid inertia is responsible for developing vortices in the expansion flow, which is trivially affected by the confinement. Fluid shear thinning causes flow separations on the contraction walls, and the interplay between the effects of shear thinning and inertia is dictated by the confinement. Fluid elasticity introduces instability and asymmetry to the contraction flow of polymers with long chains while suppressing the fluid inertia-induced expansion flow vortices. However, the formation and fluctuation of such elasto-inertial fluid vortices exhibit strong digressions from the unconfined flow pattern in a contraction–expansion microchannel of similar dimensions.

## 1. Introduction

As the earth’s conventional oil and gas resources are steadily running out, effective processes such as hydraulic fracturing to enhance the productivity of unconventional reservoirs are attracting interest lately [1]. However, in order to reduce the associated filtration loss in the process, it is necessary to comprehend the flow through a series of pore-throat structures and thus contractions and expansions [2]. Understanding the behavior of fluids with different rheological properties through such pores is also important for a wide range of other applications such as environmental remediation, extrusion, mold filling, inkjet print heads, industrial drag-reducing, and cooling processes. [3,4]. A channel (of either planar, axisymmetric, or square geometries) with an expansion and/or a contraction is one of the simplest models of these naturally occurring or industrially fabricated pores (of either nano-, micro-, or mini-sized) [5,6]. Such channels are also commonly employed in lab-on-a-chip systems to facilitate flow control [7,8], fluid mixing [9,10], particle manipulation, etc. [11,12,13]. The applications pertain to the areas of point-of-care technologies, chemical synthesis, microfluidic rheometry, etc. [14,15,16,17,18].

The expansion–contraction microchannel, referred to herein as a cavity microchannel, has been recently demonstrated to focus and isolate particles (both biological and synthetic) in a heterogeneous sample, as well as to extract pure fluids clear of particles [19,20,21]. In particular, Hur et al. [22] proposed a decade ago a high-throughput technique to enrich and isolate larger particles using the inertially formed vortices in a cavity microchannel. Since then, a number of theoretical [23,24] and experimental [25,26] studies have been reported on both the fundamentals of particle dynamics inside the cavity [27,28,29] and the biomedical applications of this microfluidic technique [30,31,32]. In a recent paper, Raihan et al. [33] demonstrated a similar vortex-based trapping and separation (by size) of particles in the flow of shear-thinning xanthan gum (XG) solution through a cavity microchannel. The operating range of Reynolds numbers in these shear-flow induced force-based methods is above a critical point, at which the flow separation occurs in the expansion–contraction region of the cavity channel because of the fluid inertial and/or shear thinning effects [23,33,34]. It is thus essential to recognize the flow regimes for fluids with different rheological properties in a cavity microchannel for a better understanding of the particle motion inside the cavity and as well for potentially broadened applications of any undisclosed interesting flow phenomena.

There have been many previous studies on the flow behaviors of different types of non-Newtonian fluids through pure contractions or expansions and contraction–expansion channels [35,36,37,38,39]. Investigations on various factors have revealed that the geometrical parameters influencing the flow patterns in such flows include the contraction (or expansion) ratio (i.e., the width of the contraction or expansion part to that of the main channel), channel aspect ratio (i.e., the width to depth ratio of the main channel), etc. [40,41,42,43,44,45]. The flow patterns of various polymer solutions such as polyethylene oxide (PEO) [46,47,48,49,50], polyacrylamide (PAA) [51,52,53], polyvinylpyrrolidone (PVP) [35], XG [35,54], DNA [55,56], and surfactant [57,58] solutions have been investigated in contraction and/or expansion microchannels to recognize both the sole and combined effects of fluid rheological properties (namely elasticity and shear thinning [59]) and inertia. Moreover, the ionic contribution has been identified through the study of elastic instabilities in the flow of hyaluronic acid (HA) sodium salt solution [60], as well as the hydrolyzed PAA solution with and without salt [61,62].

In contrast, there have been very limited studies on the understanding of non-Newtonian fluid flow in cavity microchannels. de Souza Mendes et al. [63] investigated the flow of yield stress Carbopol solutions through axisymmetric expansion–contraction ducts. They also developed a finite volume model to simulate the viscoplastic flow behavior via the generalized Newtonian liquid model. The unyielded region was found near the channel wall of the larger diameter portion with the size being a strong function of the geometrical, rheological, and flow parameters. Later, Varges et al. [64] presented an experimental study of the inertialess flow of Carbopol aqueous dispersions through annular abrupt expansions–contractions. Their observed flow patterns reveal both yielded and unyielded regions, where the yield surfaces were found to exhibit a fore-aft asymmetry in all tests because of the elastic effects. Hong et al. [65] proposed an efficient microfluidic mixer based on the inertio-elastic flow instability in PEO solution through a straight microchannel with side wells. They demonstrated an enhanced mixing via the chaotic vortices appearing in the side wells when the inertia and elasticity of the polymer solution are balanced with the Reynolds and Weissenberg numbers being both on the order of 10. The same group [66] later utilized such inertio-elastic mixing for the continuous synthesis of silica nanoparticles in a gear-shaped microchannel. Sasmal [67] conducted an extensive numerical study of the flow characteristics of a wormlike micellar solution through a long micropore with a step expansion and contraction. A special Vasquez–Cook–McKinley constitutive model [68] was used to predict the rheological behavior. Different flow regimes were identified, including the Newtonian-like, lip vortex formation, unsteady, and vortex merging regimes. More recently, Browne et al. [69] investigated the dependence of the spatial and temporal characteristics of the inertialess flow of PAA solution on the spacing between pore constrictions in a one-dimensional ordered array. They observed unstable eddies in the expansion before each constriction when the pore spacing is large. Surprisingly, the flow was observed to exhibit bistability when the pore spacing is sufficiently small. This unusual behavior was attributed to the interplay between elongation and relaxation of polymers as they are advected through the pore space.

To date, however, no work has been reported on the flow of fluids with varying rheological properties through the same cavity microchannel, thus providing a basis for a direct comparison amongst different flow parameters. Moreover, nearly none of the reported works [63,64,65,66,67,68,69] have emphasized the effect of confinement along the primary flow direction that is imposed by the expansion and contraction. Especially looking at the heavy geometric dependence of the non-Newtonian flow instabilities through the existing works mentioned above, it is essential to underline how the flow pattern in a confined geometry differs from the unconfined flow reported in single contraction and/or expansion microchannels [35,36,37,38,39,40,41,42,43,44,45,46,47,48,49,50,51,52,53,54,55,56,57,58,59,60]. We test in this work five different polymer solutions, namely PVP, XG, HA, PEO, and PAA, in their dilute/semi-dilute regimes along with Newtonian deionized (DI) water as the control experiment. The goal is to obtain a unified understanding of the effects of fluid inertia, shear thinning, and elasticity, as well as confinement. It is important to note that our tested fluids have been commonly used in microfluidic applications [13,16,17,18,70], rendering them useful to be studied directly. These fluids possess different elastic and shear thinning properties, though their infinite-shear-rate viscosity values are comparable. They are tested in a similarly wide range of flow rates to cover the effect of fluid inertia. The obtained flow regimes and vortex developments are cast into the same dimensionless parameter spaces for a quantitative comparison among the different fluids. The observed flow patterns are also compared with those of the same fluids through a planar contraction–expansion microchannel of similar dimensions [35] for a further understanding of the confinement effect.

## 2. Experiment

### 2.1. Materials

**Figure 1:**
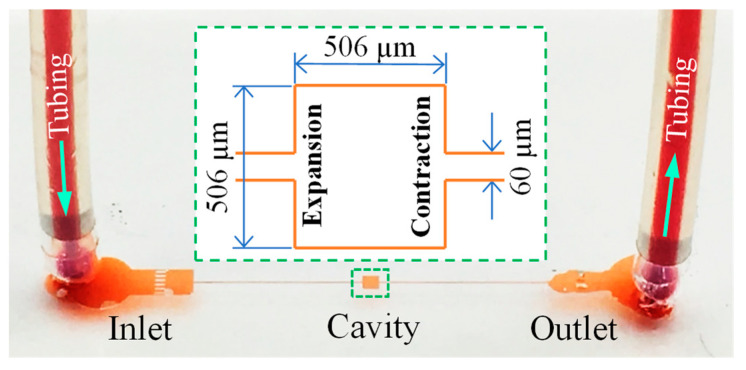
Isometric view of the fabricated cavity microchannel with the expansion–contraction dimensions displayed on the inset. The block arrows indicate the flow directions.

Figure 1 shows a photo of the sudden expansion–contraction microchannel that was fabricated with polydimethylsiloxane (PDMS) using the standard soft lithography method. The detailed procedure is describe in our previous paper [35]. The microchannel is overall 1 cm long (excluding the inlet and outlet sections for filtration purposes) with a measured width of 60 µm and height of 45 µm. It has a square-shaped cavity in the middle whose measured width is 506 µm as highlighted on the inset of Figure 1. Five types of polymer solutions were prepared from the granular powder by dissolving them into DI water (Thermo Fisher Scientific, Waltham, MA, USA) at higher weight concentrations than needed. Prior to the test, they were each diluted with DI water to the desired concentration, including: 10,000 ppm (i.e., 1 wt%.) PVP solution (with a molecular weight, $M_{w}=0.36$ MDa, Sigma–Aldrich), weakly elastic and negligibly shear thinning (i.e., Boger fluid [71]); 2000 ppm XG solution ($M_{w}\approx 2$ MDa, Tokyo Chemical Industry, Chuo-ku, Japan), negligibly elastic and strongly shear thinning [72]; 3000 ppm sodium HA solution ($M_{w}=0.357$ MDa, Lifecore Biomedical LLC, —Chaska, MN, USA), weakly elastic and mildly shear thinning [73]; 1000 ppm PEO solution ($M_{w}=2$ MDa, Sigma-Aldrich, St. Louis, MI, USA), mildly elastic and weakly shear thinning [47]; 200 ppm PAA solution ($M_{w}=18$ MDa, Polysciences, Warrington, PA, USA), strongly elastic and strongly shear thinning [74]. The control experiment was conducted with DI water.

**Figure 2:**
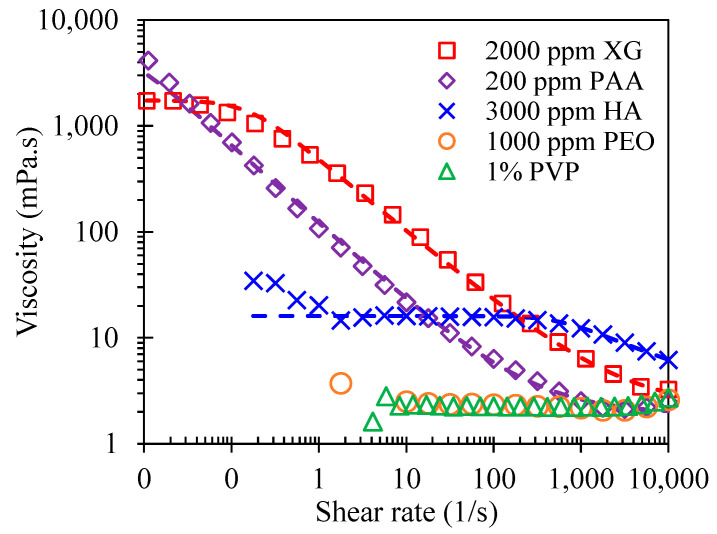
Shear viscosity data of the prepared non-Newtonian fluids, where the symbols represent experimentally measured values and the dashed lines show the fitted curves using the Carreau model for the shear thinning XG, HA, and PAA solutions.

The dynamic viscosities of the prepared non-Newtonian fluids were each measured over a wide range of shear rates with a cone-plate rheometer (Anton Paar, MCR 302, cone diameter −50 mm/angle −1°) at room temperature. The imposed duration for each value of the shear rates was kept at around 6 s. The experimental data are shown in Figure 2. The measurement was noticed to have relatively large errors for low viscosity fluids under shear rates smaller than 10 s^−1^. To quantify the shear-thinning effect of the XG, HA, and PAA solutions, we used the Carreau model [59] to curve-fit the experimental data with the smallest standard deviation,

(1) $$\frac{\eta -\eta_{\infty }}{\eta_{0}-\eta_{\infty }}={\left[1+{\left(\lambda_{C}\dot{\gamma }\right)}^{2}\right]}^{\left(n-1\right)/2}$$

where η is the fluid viscosity, $\eta_{\infty }$ is the infinite-shear-rate viscosity, $\eta_{0}$ is the zero-shear-rate viscosity, $\lambda_{C}$ is a time constant, $\dot{\gamma }$ is the fluid shear rate, and n is the power-law index. The curve fitting values of these parameters along with the rheological properties of other fluids are presented in Table 1. The relaxation times, λ, of the PVP, HA, PEO, and PAA solutions were all extracted from the literature because we were not able to obtain consistent and accurate measurements using our rheometer. Specifically, the relaxation time of 10,000 ppm PVP solution was estimated from the reported experimental value of 2.2 ms for 5% (i.e., 50,000 ppm) PVP (of equal molecular weight to the one used in this work) solution from Liu et al. [75] using the concept of effective relaxation time based upon the Zimm theory [76],

(2) $$\lambda \propto \left[\eta \right]M_{w}{\left(c/c^{*}\right)}^{3v-1}\propto M_{w}^{3v+{\left(3v-1\right)}^{2}}c^{3v-1}\propto c^{0.8}$$

where $\left[\eta \right]\propto M_{w}^{3v-1}$ is the intrinsic viscosity, v=0.6 is the quality index of a good solvent, c is the polymer concentration, and $c^{*}=1/\left[\eta \right]\propto M_{w}^{1-3v}$ is the overlap concentration for dilute polymer solutions. The relaxation time of 3000 ppm, $M_{w}=0.357$ MDa, HA solution was estimated from the reported experimental value of 0.11 ms for 700 ppm, $M_{w}=0.9$ MDa, HA solution from Haward [77] using the molecular-weight and concentration scaling suggested by the author and v=0.6,

(3) $$\lambda \propto M_{w}{}^{1.8}c^{3v-1}\propto M_{w}{}^{1.8}c^{0.8}$$

We did not consider the thixotropy [78] of the prepared non-Newtonian fluids as these polymer solutions have been commonly treated as shear thinning and viscoelastic fluids in the literature [11,12,13,16,17,18,70]. However, the occurrence of low-inertia instabilities in the flow of thixotropic fluids [79] may be an interesting direction for future work.

### 2.2. Methods

The flow in the cavity microchannel was visualized by seeding 1 µm diameter fluorescent particles (Bangs Laboratories, Fishers, IN, USA) into the prepared fluids at a volume ratio of 0.05%. A syringe pump (KD Scientific, Holliston, MA, USA) was used to drive the fluids through the channel. Plastic tubes were inserted in the inlet and outlet holes of the channel (see Figure 1). The inlet tube was connected to the pump, and the outlet tube led the fluids to a vial away from the channel. The streaklines of the tracing particles at the expansion–contraction region were recorded through an inverted microscope (Nikon Eclipse TE2000U, Nikon Instruments, Lewisville, TX, USA) with a CCD camera (Nikon DS-Qi1Mc, Lewisville, TX, USA). The exposure time of the camera was varied from 3 s at low flow rates to 0.5 s at high flow rates. The purpose of this change was to ensure that continuous streamlines can be captured in snapshot images at the expansion–contraction region of the channel. It also fulfilled the condition of keeping the number of particles tracked during the exposure time roughly similar among different flow rates. The obtained images were post-processed using the Nikon imaging software (NIS Elements AR 3.22). The fluid inertia effect on the flow pattern is characterized using the Reynolds Number,

(4) $$Re=\frac{\rho VD_{h}}{\eta \left(\bar{\dot{\gamma }}\right)}=\frac{2\rho Q}{\eta \left(\bar{\dot{\gamma }}\right)\left(w+h\right)}$$

where ρ is the fluid density (assumed to be the density of the solvent, DI water, for our dilute solutions), V is the average fluid speed in the main channel, $D_{h}$ is the hydraulic diameter of the main channel, $\eta \left(\bar{\dot{\gamma }}\right)$ is the fluid viscosity at the characteristic shear rate, $\bar{\dot{\gamma }}=2V/w$, across the width, w=60 µm, of the main channel, Q is the volumetric flow rate, and h=45 µm is the channel height. The fluid elasticity effect on the flow pattern is characterized by the Weissenberg number,

(5) $$Wi=\lambda \bar{\dot{\gamma }}=\frac{2\lambda Q}{w^{2}h}$$

The relative impact of the fluid elasticity over inertia is measured by the elasticity number,

(6) $$El=\frac{Wi}{Re}=\frac{\lambda \eta \left(\bar{\dot{\gamma }}\right)\left(w+h\right)}{\rho w^{2}h}$$

which is independent of fluid kinematics except for shear-thinning fluids. Note that the values of dimensionless Re, Wi, and El (at 10 mL/h, see Table 1) presented in this work were all estimated under the inlet conditions for each prepared solution. For the ease of description, a fluid is regarded here as weakly elastic for 0<El<1, mildly elastic for 1≤El<10, and strongly elastic for El≥10. The fluid shear-thinning effect on the flow pattern is characterized by the power-law index, n, in Table 1, where n<0.5 is regarded here as strongly shear thinning, 0.5≤n<0.75 as mildly shear thinning, and 0.75≤n<1 as weakly shear thinning.

## 3. Results

### 3.1. Fluid Rheological Effects on the Cavity Flow

#### 3.1.1. DI Water: Effects of Fluid Inertia

**Figure 3:**
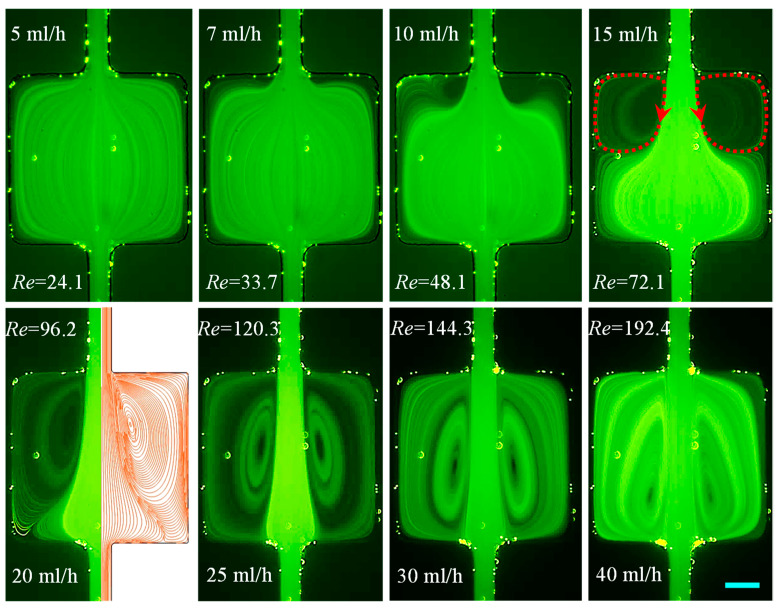
Flow of Newtonian water (downward) at the expansion–contraction region of the cavity microchannel. Fluid circulations are formed only at the expansion part (highlighted by the dashed-line arrows on the **top-right** image) and no vortices are observed on the contraction walls for the flow rates tested. The right half of the **bottom-left** image shows the simulated fluid streamlines under the corresponding experimental conditions. The scale bar represents 100 µm.

Since water is an inelastic fluid with a constant viscosity, it provides a good base to investigate only the inertial effect. We tested the water flow in the cavity microchannel with Re spanning nearly two orders of magnitude from 2.4 (at 0.5 mL/h) to 216.5 (at 45 mL/h). As viewed from the particle streaklines in Figure 3, the flow is symmetric about the channel centerline without any bending or vortices at the flow rates smaller than 5 mL/h. A pair of small-lip vortices forms on the expansion walls of the channel as the flow rate reaches around 7 mL/h (Re=33.7). With the increase in flow rate, these vortices grow to extend to the side walls of the cavity at about 15 mL/h, yielding the so-called corner vortices whose size increment then gets restricted to only the channel length direction. At 20 mL/h, the symmetric corner vortices touch the contraction walls as their cores move downstream throughout the process, which matches well with the numerically predicted fluid streamlines from a three-dimensional model in COMSOL^®^. Each vortex assumes the entire half-cavity (i.e., cell vortex) at 30 mL/h confining the bounds of the primary flow to be almost the same as that in the main channel. Further increasing the flow rate shifts the cores of the vortices further towards the contraction walls because of the enhanced fluid inertia. However, the size of the cavity places an upper limit upon the vortex length. Note that the tracer particles failed to follow the streamlines and enter the inertial vortices in some of the cases (e.g., 10 mL/h and 15 mL/h), leaving dark spots on the images of Figure 3. We attribute this phenomenon to the potential separation of particles at the re-entrant corners of the expansion part because of perhaps the combined influence of inertial and centrifugal effects.

#### 3.1.2. PVP Solution: Effects of Fluid Elasticity Along with Inertia

**Figure 4:**
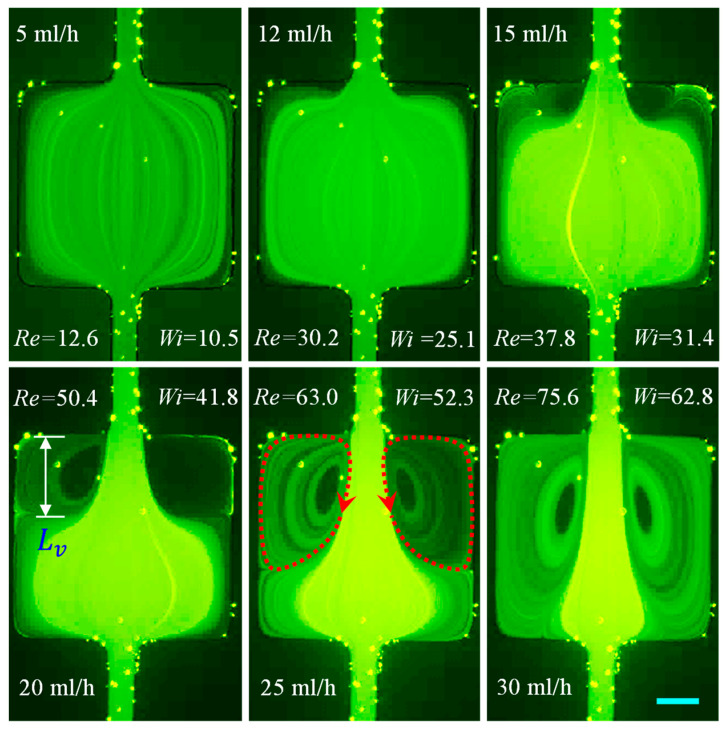
Flow of weakly elastic and negligibly shear thinning PVP solution (downward) at the expansion–contraction region of the cavity microchannel. Fluid vortices (with a measured length, $L_{V}$, to be used in Figure 10) are developed only in the expansion flow (highlighted by the dashed-line arrows on **bottom-middle** image), and no disturbances are observed in the contraction flow for the span of the flow rates tested. The scale bar represents 100 µm.

The prepared PVP solution is a weakly elastic Boger-like fluid (i.e., n≅1) with El=0.83. We tested this solution for the flow rate of up to 35 mL/h (Re=88.2) to study both the sole effect of fluid elasticity and its combined effect with fluid inertia. We were unable to reach values of Re comparable to those in the water flow because of the greater fluid resistance in the PVP solution. Figure 4 shows the flow development at the expansion–contraction region of the cavity microchannel. The presence of fluid elasticity does not apparently affect the flow pattern as the inertia-induced fluid streamlines in DI water (Figure 3) are visually similar to those in the PVP solution, except for the slightly earlier formation of vortices in the latter at approximately Re=30. This observation appears to be consistent with our previously reported similarity between the inertial flows of water and 5% PVP solution (El=17.2, strongly elastic) in a contraction–expansion microchannel of similar dimensions, which was attributed to the short-chain structure of PVP molecules [35]. The confinement does not induce any disturbances to the cavity flow other than just restricting the growth of the vortices, similar to that in the water flow in Figure 3. A comparison of the channel lengthwise dimensions of the corner vortices, $L_{v}$ (highlighted on the image in Figure 4), between water and the PVP solution as well as other tested fluids, is presented in Section 3.3.

#### 3.1.3. XG Solution: Effects of Fluid Shear Thinning Along with Inertia

**Figure 5:**
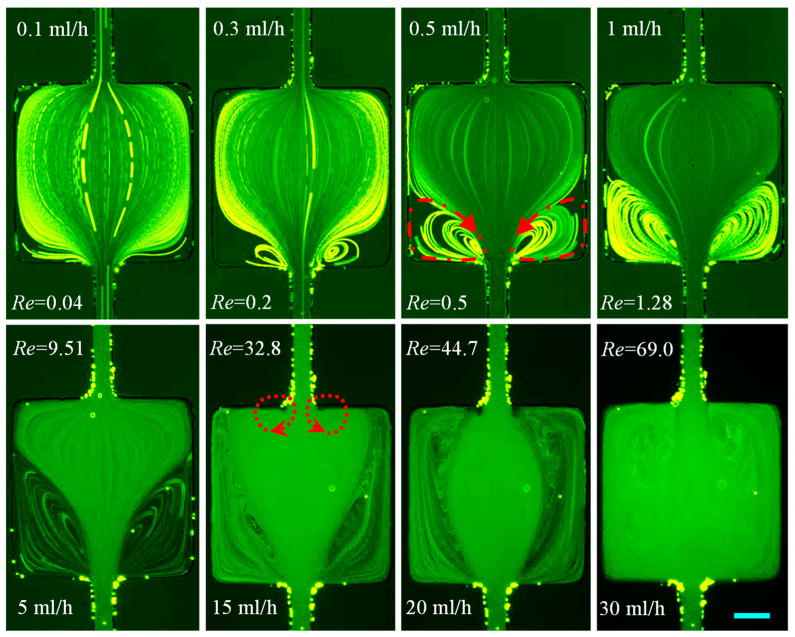
Flow of negligibly elastic and strongly shear thinning 2000 ppm XG solution (downward) at the expansion–contraction region of the cavity microchannel. Fluid vortices are developed in a creeping contraction flow (Re < 1, highlighted by the dashed-dotted-line arrows on the **top-row** image) because of the fluid shear thinning effect and later also in the inertial expansion flow (highlighted by the dashed-line arrows on the **bottom-row** image). The scale bar represents 100 µm.

The prepared XG solution (n=0.33) is a strongly shear-thinning fluid with a minimal elasticity [80]. It therefore serves as a good fluid to demonstrate the effect of shear thinning and its combined effect with inertia on the cavity flow. We can see in Figure 5 that small symmetric lip vortices are already developed on the contraction walls of the cavity at a flow rate of 0.1 mL/h (Re=0.04≪1), which is likely a consequence of the fluid shear thinning effect. These lip vortices soon turn into corner vortices before Re reaches the value of Re=0.5 at 0.5 mL/h. The secondary flow inside the vortices is, however, much slower than the primary flow passing through the centerline region of the microchannel. The growth in the circulation size along the channel length continues until Re~10 at 5 mL/h while maintaining the symmetry about the channel centerline. After this point, the intensity of the secondary flow rises without further increment in the vortex length. The cores of the fluid vortices shift upwards, and this state is maintained until *Re* reaches 32.8 at 15 mL/h. The fluid inertia takes effect at this point as small symmetric lip vortices are formed at the re-entrant corners on the expansion walls. Simultaneously, each of the shear thinning-induced contraction flow vortices extends to the expansion walls on their respective sides and then merges with the small inertia-induced expansion flow vortices at 20 mL/h. The shape of these merged vortices as a result of the combined fluid shear thinning and inertial effects is different from that of the inertially formed vortices in the Newtonian water (Figure 3) or PVP solution (Figure 4). Further increasing the flow rate causes the relocation of the cores to near the expansion walls at 30 mL/h (Re=69.0). The flow becomes chaotic afterward because of perhaps the strongly interacted contraction and expansion flows in a confined cavity. This is different from the maintained symmetric vortices surrounding the unconfined primary flow in a constriction microchannel even at Re>100 [35].

#### 3.1.4. HA Solution: Effects of Weak Elasticity and Mild Shear Thinning Along with Inertia

**Figure 6:**
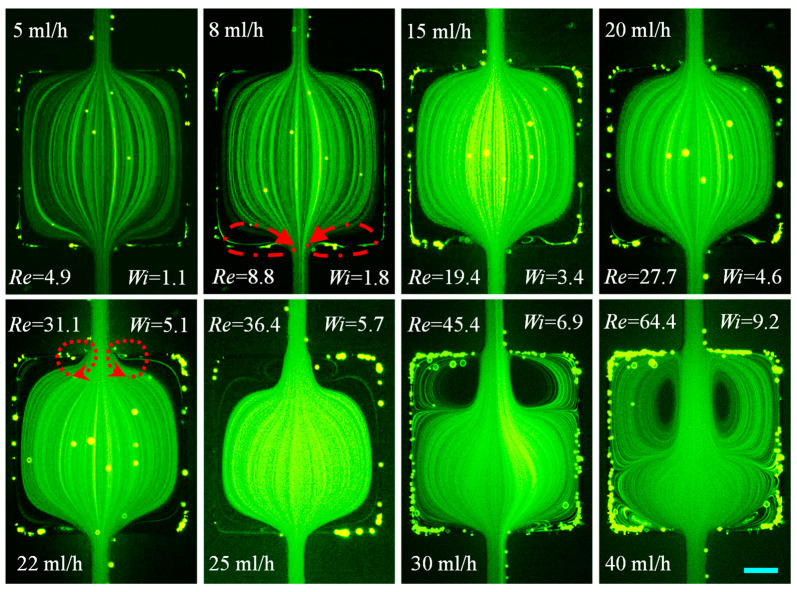
The flow of weakly elastic and mildly shear-thinning 3000 ppm HA solution (downward) at the contraction–expansion region of the cavity microchannel. Fluid vortices are first developed in the inertial contraction flow (highlighted by the dashed-dotted-line arrows on the **top-row** image) because of the dominant shear thinning effect over the elasticity effect and then also in the inertial expansion flow (highlighted by the dashed-line arrows on the **bottom-row** image). The scale bar represents 100 µm.

**Figure 7:**
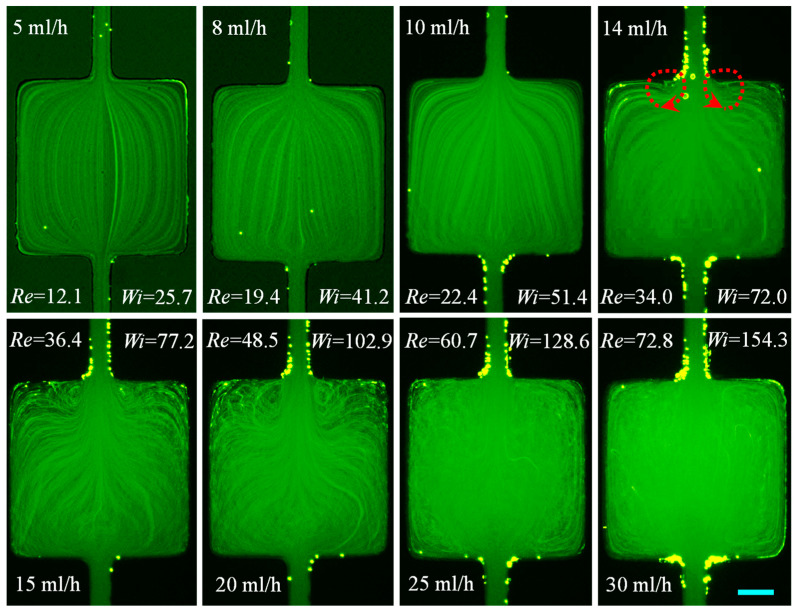
Flow of mildly elastic and weakly shear thinning 1000 ppm PEO solution (downward) at the contraction–expansion region of the cavity microchannel. Fluid circulations are formed on the expansion walls (highlighted by the dashed-line arrows on the **top-right** image) at a similar value of Re to that in the flow of water (Figure 3) and PVP solution (Figure 4). They grow and become chaotic with the increase in flow rate. The scale bar represents 100 µm.

The prepared HA solution is a weakly elastic (El=0.20) and mildly shear thinning (n=0.62) fluid. As viewed from the images in Figure 6, the low Re (≤4.9) flow of HA solution exhibits symmetric cavity-shape conforming streamlines with no sign of fluid bending or vortices. At the flow rate of 8 mL/h, a pair of small-lip vortices develop at the re-entrant corners on the contraction walls because of the fluid shear thinning effect. They do not seem to arise from the fluid elasticity effect as no such vortices are observed in the viscoelastic PVP (Figure 4) or PEO (Figure 7) solution with a negligible-to-weak shear thinning effect. The corresponding value of Re=8.8 is, however, more than two orders of magnitude higher than that observed in the XG solution (Figure 5). This phenomenon may indicate a strong influence of both the shear thinning extent and the polymer structure because the HA polymer has a much shorter chain than the XG polymer and hence causes less extensional stretching and reorientation. The flow separation at Re=31.1 (22 mL/h) initiates the onset of Newtonian-like lip vortices on the expansion walls, which grow on to reach the sidewalls of the cavity and become corner vortices at Re=45.4 (30 mL/h). In contrast, the contraction-wall lip vortices grow very slowly with the increase of flow rate after formation because of perhaps the short chain of HA polymers. Only at the flow rate of 40 mL/h with Re=64.4 do the shear-thinning-induced contraction-wall vortices finally reach the salient corners. Concurrently, the inertia-induced expansion-wall vortices further extend downwards and almost merges/interacts with the contraction-wall vortices because of the confinement. Such a nearly independent existence of the expansion and contraction flow vortices exhibits a similarity to the unconfined flow of HA solution in a constriction microchannel [50].

#### 3.1.5. PEO Solution: Effects of Mild Elasticity and Weak Shear Thinning Along with Inertia

The prepared PEO solution is a mildly elastic (El=2.12) and weakly shear thinning fluid with a similar viscosity to the weakly elastic and negligibly shear thinning PVP solution. One key difference between these two fluids lies in the longer polymer chain of the PEO molecule and hence the stronger elasticity effect of its solution. Figure 7 shows the flow of the PEO solution through the cavity microchannel for flow rates ranging from 5 mL/h (Re=12.1) to 30 mL/h (Re=72.8). No apparent deviations from the symmetric Newtonian flow streamlines (Figure 3) are observed at 5 mL/h. A diverging flow pattern appears in the contraction part of the cavity at 8 mL/h (*Re* = 19.4) and becomes significantly stronger with the increase of flow rate. In the expansion part of the cavity, bending streamlines start taking the form of two small vortices at the re-entrant corners at 14 mL/h. The corresponding value of Re=34.0 is close to that in the flow of water and PVP solution for the onset of inertial expansion-flow vortices. However, the vortices in the PEO solution are not laminar in nature like in the other two fluids. The chaotic behavior of the contraction flow increases with flow rate as the lip vortices of the expansion flow evolve to approach the salient corners of the expansion walls at 20 mL/h (Re=48.5). The chaos completely takes over the flow pattern, and any shape of vortices become visually unrecognizable when the flow rate reaches 30 mL/h. Despite having a similar viscosity and a similar order of fluid elasticity (El~1) to that of the PVP solution (Figure 4), the streamline images are completely different in the PEO solution (Figure 7). The reason perhaps lies in other rheological parameters than just the relaxation time such as the extensional viscosity or the wall adsorption/depletion dynamics of the polymers here, which in turn would affect the wall shear rate and thus the overall rheological response of the flow. More interestingly, we have previously encountered single or asymmetric vortices in the contraction flow and no vortices in the expansion flow of PEO solutions in a constriction microchannel [35,50], which is opposite to the flow patterns in the cavity microchannel (Figure 7). The question that ensues from these observations is whether the flow of non-Newtonian fluids through a cavity or constriction microchannel can be each viewed as a simple superposition of the contraction and expansion flows.

#### 3.1.6. PAA Solution: Effects of Strong Elasticity and Strong Shear Thinning and Inertia

**Figure 8:**
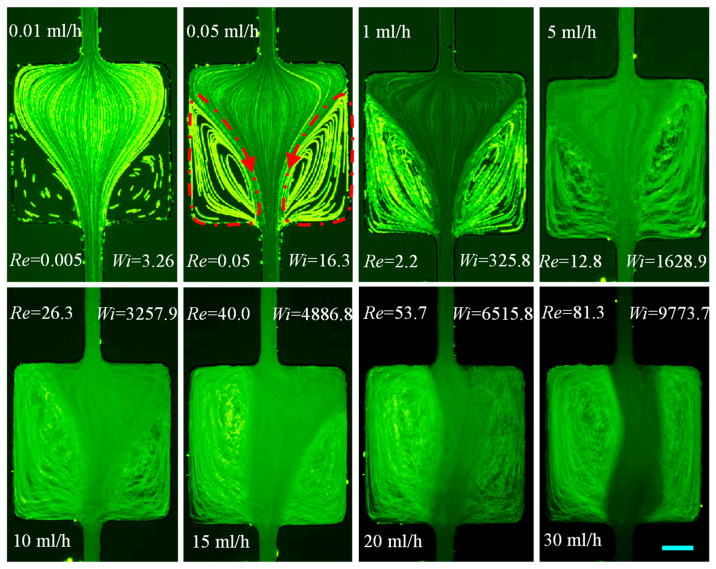
Flow of strongly elastic and strongly shear thinning 200 ppm PAA solution (downward) at the expansion–contraction region of the cavity microchannel. Fluid vortices are developed in a nearly inertialess contraction flow (Re≪1, highlighted by the dashed-dotted-line arrows on the **top-row** image) because of the strong shear thinning and the long polymer chains. No inertia-induced expansion flow vortices are observed for the tested flow rates because of the suppression of the strong fluid elasticity. The scale bar represents 100 µm.

**Figure 9:**
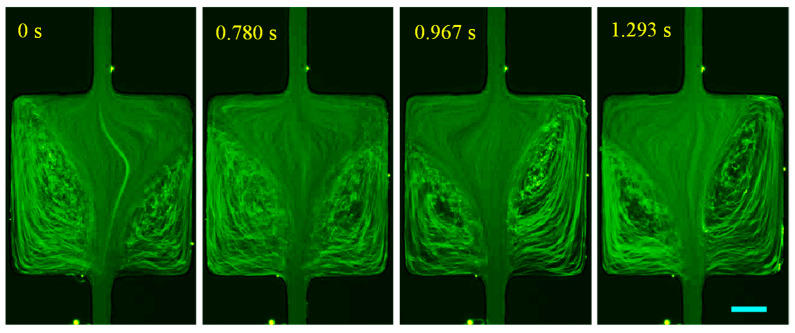
Sequential images showing the oscillation of vortices in the cavity flow of strongly elastic and strongly shear thinning 200 ppm PAA solution (downward) at the flow rate of 5 mL/h. The scale bar represents 100 µm.

**Figure 10:**
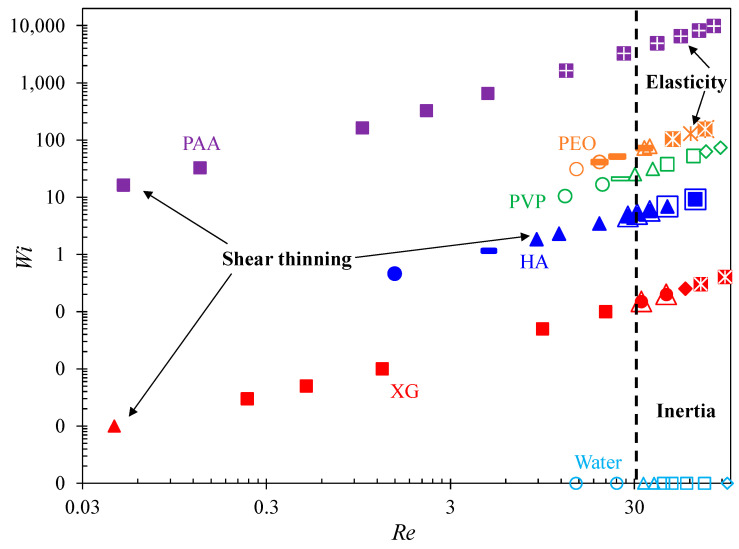
Summary of flow regimes in the Re−Wi space for the cavity flow of Newtonian water and non-Newtonian polymer solutions. Filled and hollow markers are, respectively, for the contraction and expansion flows through the microchannel: circles for no bending streamlines or vortices; rectangles for bending streamlines; triangles for stable lip vortices; squares for stable corner vortices; diamonds for stable symmetric vortices completely extended between the expansion and contraction walls (i.e., cell vortices); crosses are for unstable asymmetric vortices; asterisks are for unstable chaotic cell vortices. The vertical dashed line at Re=30 marks the transition to the flow regime that fluid inertia plays an important role as the inertia-induced vortices start occurring at the re-entrant corners of the expansion flow. Note that the Weissenberg number has been assumed to be 0.0001 for pure water (at any flow rates) and XG solution (at 0.01 mL/h) for the purpose of graphing.

The prepared PAA solution is a strongly elastic (El~123.64) and strongly shear-thinning (n=0.25) fluid with a non-negligible second normal stress difference that can induce a secondary flow even in a straight uniform microchannel [81]. The cavity flow of the PAA solution is shown in Figure 8 for Re spanning over four orders of magnitude. Large symmetric corner vortices are observed on the contraction walls under a nearly inertialess flow condition of Re=0.005≪1 (0.01 mL/h), significantly lower than the value of Re=0.5 in the XG solution with a roughly equal power-law index. This phenomenon may indicate a much stronger influence of the polymer structure than the fluid elasticity as PAA molecules have much longer chains than the XG solution. An increase in flow rate to 1 mL/h (Re=2.2) only extends slightly the length of the still symmetric vortices inside the cavity while the secondary flow therein is intensified. Further increasing the flow rate to 5 mL/h (Re=12.8) renders the circulations to become asymmetric. Moreover, the two vortices are observed to oscillate without any apparent period at this flow rate (see, for example, the sequential images in Figure 9) and 10 mL/h (Re=26.3) as well. The fluid inertia-induced expansion wall vortices at around the value of Re=30, which appear in the flow of both XG (Figure 5) and HA (Figure 6) solutions, are unable to differentiate from the unstable contraction flow. Progressing to higher flow rates seems to diminish the fluid oscillations, which is counterintuitive and opposite to the observation in the constriction microchannel [35]. Such a phenomenon could be the result of a combined effect of local shear rate and polymer gelation under certain conditions enacted by the confinement. However, a thorough investigation is in order in this direction.

### 3.2. Summary of the Cavity Flow Pattern

A summary of the observed flow regimes for the prepared fluids is plotted in the Re−Wi space (with a log-log scale) and shown in Figure 10. The Weissenberg number for the DI water is assigned to a constant small value, Wi=0.0001 for the purpose of plotting in the logarithmic scale. This same value of Wi is also assigned to the XG solution at 0.01 mL/h, based on which Wi at higher flow rates can be estimated using Equation (5). For the contraction flow (see the filled symbols in Figure 10), the strongly shear-thinning XG and PAA solutions both exhibit large symmetric stable vortices in the creeping flow regime (Re<1), which grow significantly with the increase in flow rate and become chaotic or unstably asymmetric under large flow rates. The contraction flow of the mildly shear-thinning HA solution also shows vortices, which, however, develop at a much greater value of Re=10 (approximately) and with a much smaller circulation size. At an even larger value of Re=20 (approximately), the contraction flow of weakly shear-thinning PEO solution displays diverging streamlines, which grow into chaos under high flow rates and never convert into vortices. In contrast, the contraction flow of constant-viscosity water and PVP solution do not demonstrate any sign of flow separation or streamline bending for all the tested Re values.

For the expansion flow, all the prepared fluids except the PAA solution demonstrate flow separation from the cavity walls at around a similar value of Re=30. We thus highlight this Re on the plot in Figure 10 (see the vertical dashed line) as the onset of fluid inertia-induced expansion flow vortices and hence the transition to the flow regime that inertia plays an important (and even dominant in some fluids) role. For water and PVP solution, the expansion flow remains stable in the whole range of tested Re values. Fluid vortices appearing from the re-entrant corners of the expansion walls reach the salient corners and then completely occupy each side of the cavity. A similar development is observed for the expansion flow of HA solution as well, but the tested Re is not high enough for the fluid inertia-induced expansion-flow vortices to reach the fluid shear-thinning induced contraction-flow vortices in our tests. The expansion flow vortices in the PEO and XG solutions are different from those in the water, PVP, or HA solution as they remain as lip vortices and do not grow much in size before the contraction flow in the cavity turns chaotic at a moderate value of Re~60. This is because the contraction flow of these two fluids becomes very strong with the increase of fluid inertia inside the confined cavity as mentioned above. No vortices are observed in the expansion flow of PAA solution as the contraction flow vortices almost occupy individually both side wells of the cavity and become unstable at Re~10.

### 3.3. Summary of the Vortex Development

**Figure 11:**
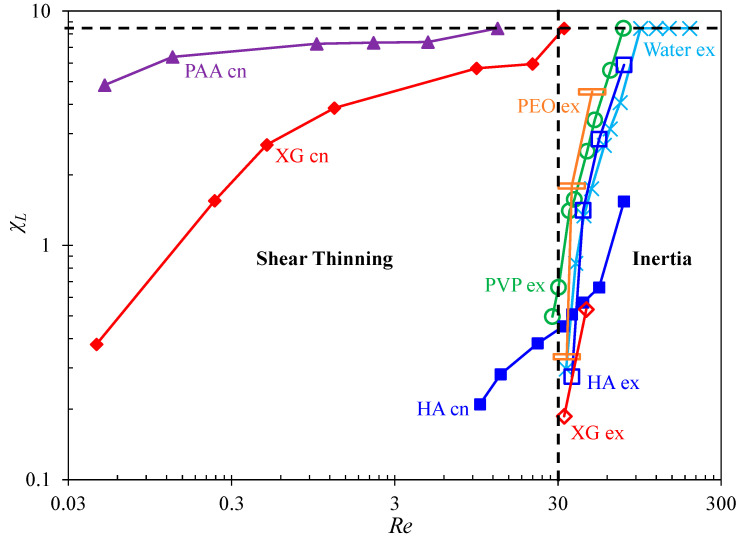
Summary of the vortex length (normalized by the microchannel width), $\chi_{L}=L_{v}/w$, against the Reynolds number, Re, in the cavity microflow of Newtonian water and non-Newtonian polymer solutions. Filled and hollow markers are for the vortices in the contraction and expansion parts of the microchannel, respectively. The horizontal dashed line at $\chi_{L}=8.4$ marks the maximum vortex length as the size of the cavity is met by the vortices (i.e., cell vortices). The vertical dashed line at Re=30 marks the onset of the fluid inertia-induced vortices at the re-entrant corners of the expansion flow. Note that only the size of the stable vortex (either symmetric or asymmetric) is shown in this plot.

The vortex development is characterized by the vortex length, $L_{v}$ (see the highlighted dimension in Figure 4), which can be measured from the experimental images (i.e., Figure 3, Figure 4, Figure 5, Figure 6, Figure 7 and Figure 8) at the contraction and/or expansion parts of the cavity microchannel. Figure 11 shows the growth of the normalized vortex length, $\chi_{L}=L_{v}/w$, with the increase in Re in each of the prepared fluids. Vortices form at very small values of Re≪1 in the contraction flow of strongly shear thinning XG and PAA solutions. Their curves in the $Re-\chi_{L}$ plot show similar patterns, with an incremental trait at the beginning followed by a plateau of no apparent growth with change in Re. They regain their incremental aspects after that and reach the maximum, where the vortices are inhibited from any further growth as the size of the cavity is met in length (see the horizontal dashed line, $\chi_{L}=8.4$, in Figure 11), at Re=12.8 in the PAA solution and Re=32.8 in the XG solution, respectively. The contraction flow vortices in the mildly shear thinning HA solution commence at a much higher value of Re=8.8 and have a much smaller size than in the XG and PAA solutions. These observed trends appear to be consistent with the decreasing shear thinning effect (as well as the decreasing polymer molecular weight) of the PAA, XG, and HA solutions in that order.

The vortices developed from the expansion part of the cavity originate at a similar value of Re=30 in all the prepared fluids except for the PAA solution, as seen from the vertical dashed line in Figure 11. The increasing pattern of $\chi_{L}$ with Re that follows is steeper and more straightforward than that of the fluid shear thinning-induced contraction flow vortices. Noticeably, the curves for these cases fit closely together in the plot. Since the expansion flow vortices are typical in the inertial flow of Newtonian fluids, the overlapping of the curves confirms that such vortices in the polymer solutions here are also (primarily) consequences of inertial effects in the cavity flow. At high values of Re>75, the fluid inertia-induced expansion-flow vortices can reach the size limit of the cavity (see the horizontal dashed line, $\chi_{L}=8.4$, in the plot) in water, PVP, and HA (if the flow rate is further increased) solutions. They are, however, absorbed by the strong chaotic contraction flows in both the PEO and XG solutions and disappear before Re reaches the order of 60. No expansion flow vortices are observed in the PAA solution as the contraction-flow vortices occupy the entire cavity well ahead of Re=30.

## 4. Conclusions

We have experimentally studied the flow of five types of non-Newtonian polymer solutions and compared them with that of water in a planar expansion–contraction microchannel. The tests were performed with a wide range of Reynolds and Weissenberg numbers, from which the sole and combined effects of fluid inertia, shear thinning, and elasticity are demonstrated under the confined condition in the cavity. The results are plotted in the non-dimensional Re−Wi and $Re-\chi_{L}$ spaces for the flow regime and vortex development, respectively. We have also compared the experimental observations with the unconfined flow of the same fluids in a planar contraction–expansion microchannel of similar dimensions. In sum, fluid inertia induces circulations in the expansion flow of water, which is trivially impacted by the confinement as the expansion flow circulations are merely restricted from growing at the contraction walls without showing any other significant event or instability. Fluid shear thinning causes the formation of stable symmetric vortices in the contraction flow of the XG solution, which interact with the expansion flow vortices inside the cavity destabilizing the flow. Fluid elasticity does not draw any disturbances to the contraction or expansion flow of the PVP and PEO solutions unless the polymer has long chains and the fluid inertia takes effect. The flow in the PEO solution becomes chaotic without showing any contraction flow vortices, which deviates from the unconfined constriction flow. The weak presence of shear thinning and elasticity in the HA solution barely affects the independent existence of the contraction and expansion flow vortices. In contrast, the combination of strong shear thinning and strong elasticity in the PAA solution surprisingly stabilizes the fluctuations of the contraction flow vortices at high inertia. Such stabilization is not observed in the unconfined flow of the same fluid through a constriction microchannel. Hence, confinement plays a key role in determining the extensional flow instabilities of polymer solutions. In future work, we will study the flow of various non-Newtonian fluids [82,83] through microfluidic porous media models with a 1D [84,85] or 2D [61,86] array of cavities and constrictions. The goal would be to see how a flow through several cavities, both in rows/columns/arrays and randomly disposed, would behave. This would allow us to see how they interact or interfere with each other and if any of the effects presented here are confirmed on a larger scale or if other new dynamics come up. It is hoped that our experimental results in this and future work will provide useful data for the validation of theoretical studies on such flows in terms of appropriate constitutive models (e.g., Oldroyd-B [87] and FENE-P [88,89] models).

## Figures and Tables

**Table 1:** Rheological properties of the prepared fluids. The elasticity number, El, was estimated at a flow rate of 10 mL/h for all the fluids.

Solution	$\eta_{0}\;(\mathrm{mP}\cdot s)$	$\eta_{\infty }\;(\mathrm{mPa}\cdot s)$	$\lambda_{C}\;(s)$	n	λ (ms)	El
DI Water	1	1	−	1	0	0
10,000 ppm PVP	2.1	2.1	−	≅1	0.61	0.83
2000 ppm XG	1740	1.8	6.6	0.33	~0	~0
3000 ppm HA	16	1.5	0.0018	0.62	0.067	0.20
1000 ppm PEO	2.18	2.18	−	~1	1.5 *	2.12
200 ppm PAA	4900	2.1	151	0.25	95 **	123.64

* Rodd et al. 2005 [46]; ** Poole and Escudier 2004 [74].
